# Granulomatosis With Polyangiitis: A Clinical Case

**DOI:** 10.7759/cureus.32410

**Published:** 2022-12-11

**Authors:** Filipa Rodrigues, Ana Isabel Oliveira Sá, Marta Mendes, Eduardo Macedo, Isabel Apolinário

**Affiliations:** 1 Internal Medicine, Hospital of Braga, Braga, PRT

**Keywords:** hemoptysis, clinical case, acute kidney injury, anti-neutrophil cytoplasmic antibody, vasculitis

## Abstract

Anti-neutrophil cytoplasmic antibody (ANCA)-associated vasculitis is characterized by inflammation and the destruction of small- and medium-caliber blood vessels in the presence of circulating ANCAs. Anti-neutrophil cytoplasmic antibody-associated vasculitis predominantly affects the lung and kidney with a multifactorial pathogenesis. This case refers to a 55-year-old woman with constitutional symptoms, hypoacusis, cough, and bloody sputum. Physical examination revealed polypnea and decreased lung auscultation at the bases; blood work showed elevated inflammatory parameters, acute kidney injury, and hematuria; pulmonary computed tomography revealed areas of peribronchial thickening in both lungs; immunological study resulted in positive ANCA-PR3. Corticotherapy and double immunosuppression with cyclophosphamide and rituximab were initiated, which resulted in clinical and analytical improvement. This case of granulomatosis with polyangiitis (GPA), with pulmonary, renal, cutaneous, and ear involvement, allows us to demonstrate the importance of timely clinical suspicion and initiation of immunosuppression for a favorable disease prognosis.

## Introduction

Anti-neutrophil cytoplasmic antibody (ANCA)-associated vasculitis is an uncommon systemic autoimmune disease with an incidence of about 20 per million inhabitants in Europe and North America. It is predominantly observed in males, and its incidence increases with age, peaking between 60 to 70 years [1,2].

Anti-neutrophil cytoplasmic antibody-associated vasculitis predominantly affects small- and medium-caliber blood vessels and is divided into three clinical entities: granulomatosis with polyangiitis (GPA), microscopic polyangiitis, and eosinophilic granulomatosis with polyangiitis. The ANCA serotypes can be identified as proteinase 3 (PR3) which is most commonly associated with GPA (75%), and myeloperoxidase (MPO) which is most commonly associated with MPA (60%), or vasculitis limited to the kidneys (80%) [1,2,3].

The exact mechanisms leading to excess ANCA production are not well understood, and various factors have been implicated in its pathogenesis, including environmental, genetic, and infectious factors [1,2].

Neutrophils are the main mediators of vascular injury, and under certain conditions (e.g., a response to infection or inflammation), they undergo a “priming” process in which they display target antigens, such as PR3 and MPO, on their surface membranes. The ANCAs bind to these autoantigens which results in the excessive activation of neutrophils that adhere to the vascular endothelium. This hyperactivation is followed by abnormal cytokine production and the release of reactive oxygen species and proteases, both of which mediate vascular injuries. Activated neutrophils also undergo necroptosis in which neutrophil extracellular traps (NETs) are released to sustain the activation of the alternative complement pathway and leads to the direct injury of the endothelium. Furthermore, NETs contain PR3 and MPO, and their chronic elevation leads to their recognition by dendritic cells and subsequently by T cells and plasma cells as neoantigens. Therefore, the continuous production of ANCA-PR3 and MPO from lymphocytes results in a cycle of neutrophil hyperactivation, inflammatory activity, and vasculitis [1,2,3].

## Case presentation

A 55-year-old woman with a personal history of obesity and arterial hypertension who had been taking perindopril was referred to the emergency service (ER). Her symptoms included an irritative cough, sleep hyperhidrosis, polydipsia, asthenia, and anorexia with 15 days of evolution associated with abdominal pain, fever, and hemoptysis expectoration with three days of evolution. She had been previously medicated with azithromycin for presumed respiratory infection without improvement of the clinical picture. She had also used the ER several times in the previous month due to auditory symptoms (otalgia, hypophonia, and tinnitus), for which she was medicated with systemic corticotherapy and topical antibiotic therapy without resolution of the clinical picture. A physical examination indicated that she was pale and dehydrated. She presented with xerostomia and polypnea without respiratory insufficiency, arthralgia, or cutaneous alterations. She was hemodynamically stable with a reduced vesicular murmur at both bases on pulmonary auscultation.

**Figure 1:**
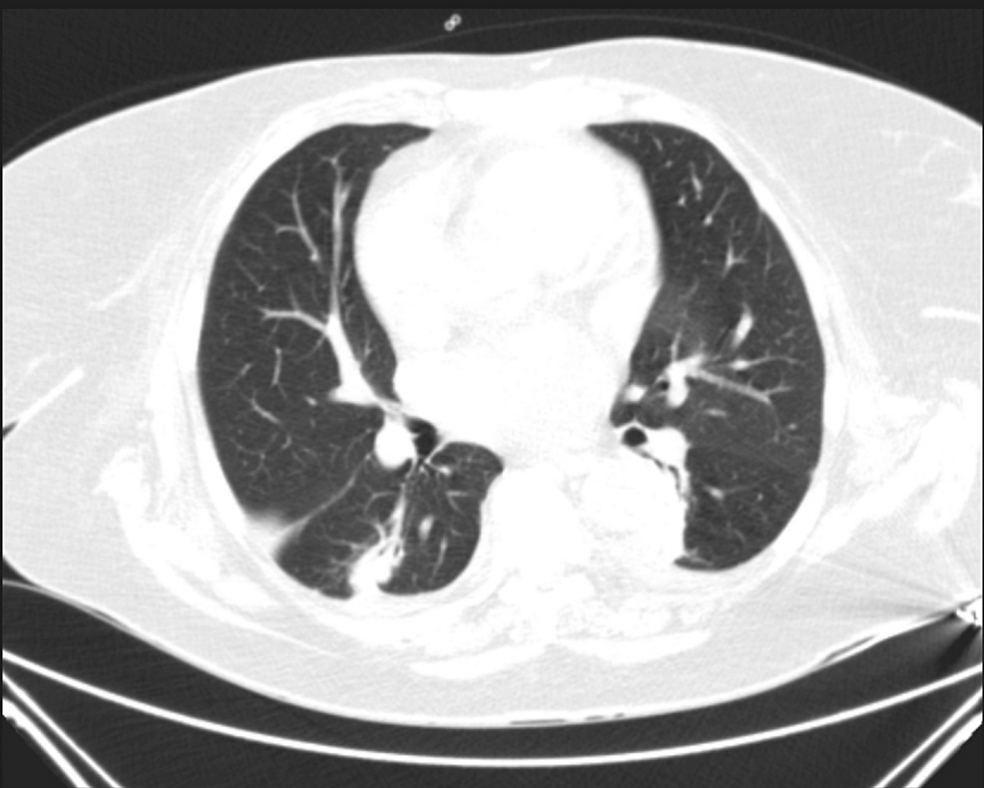
Chest computed tomography showing irregular peribronchial thickening areas and pericentimetric nodular lesions

Blood work revealed normocytic normochromic anemia, leukocytosis with neutrophilia, and high levels of C-reactive protein, urea, and creatinine. A urine summary revealed proteinuria and erythrocyturia (Table 1). Thoraco-abdominal computed tomography indicated areas of irregular peribronchial thickening and pericentimetric nodular lesions (Figure 1), suggesting inflammatory bronchiectasis and slight asymmetric densification of left perirenal fat. Accordingly, the patient was admitted to the hospital with a presumed diagnosis of non-obstructive pyelonephritis with acute renal injury and infected bronchiectasis. Empirical antibiotic therapy with ceftriaxone and clarithromycin was initiated.

**Table 1:** Biochemical testing Hgb: Hemoglobin; WBC: White blood cells; AST: Aspartate transaminase; ALT: Alanine transaminase; CRP: C-reactive protein; ESR: Erythrocyte sedimentation rate; PT: Prothrombin time; PTT: Partial thromboplastin time; INR: International normalized ratio; Comp: Complement; GBM: Glomerular basement membrane; ANA: Antinuclear antibody; ANCA-PR3: Anti-neutrophil cytoplasmic antibody -proteinase-3; RBC: Red blood cell; LE: Leukocyte esterase; UPCR: Urine protein creatinine ratio

Test	Result	Normal Range	Test	Result	Normal Range
Hgb	8.8 g/dL	11.9-15.6	ESR	103mm/hr	<15
WBC	9.5 x 10^3^/microL	4.1-10.8	PT	14.5 seconds	8-14
Platelets	320 x 10^3^/microL	150-400	PTT	29.3 seconds	25-37
Neutrophil %	86.7%	34.0-69.5	INR	1.25	0.8-1.2
Sodium	131 mmol/L	136-145	Comp C3	175 mg/dL	90-180
Potassium	4.1 mmol/L	3.5-5.1	Comp C4	33 mg/dL	10-40
Chloride	97 mmol/L	98-107	GBM-antibody	0	<40
Bicarbonate	17.3 mmol/L	21-26	ANA	Negative	Negative
Total calcium	6.9 mg/dL	8.3-10.6	ANCA-MPO Antibody	0,1 U/mL	<10
Urea	181 mg/dL	19-49	ANCA-PR3 Antibody	>1067,0 U/mL	<20
Creatinine	7.6 mg/dL	0.6-1.2	Urine Blood	Large	Negative
Glucose	98 mg/dL	70-110	Urine LE	Large	Negative
Protein	4.9 g/dL	5.7-8.2	Urine Nitrite	Negative	Negative
Albumin	2.7 g/dL	3.4-5.0	Urine RBC	>50/HPF	0-2
AST	20 units/L	12-40	Urine WBC	10-25/HPF	None
ALT	21 units/L	7-40	Uroculture	Negative	Negative
Total Bilirubin	0.18 mg/dL	0.3-1.2	UPCR	8 mg/mg	<0.5
CRP	310 mg/L	<5			

Throughout hospitalization the patient maintained daily febrile peaks even under antibiotic therapy, thus initiating respiratory failure requiring supplementary oxygen therapy. She also exhibited a progressive worsening of renal function, reaching a maximum value of 9.3 mg/dl of creatinine, requiring renal replacement therapy and a transfusion of two units of erythrocyte concentrate due to worsening anemia.

**Figure 2:**
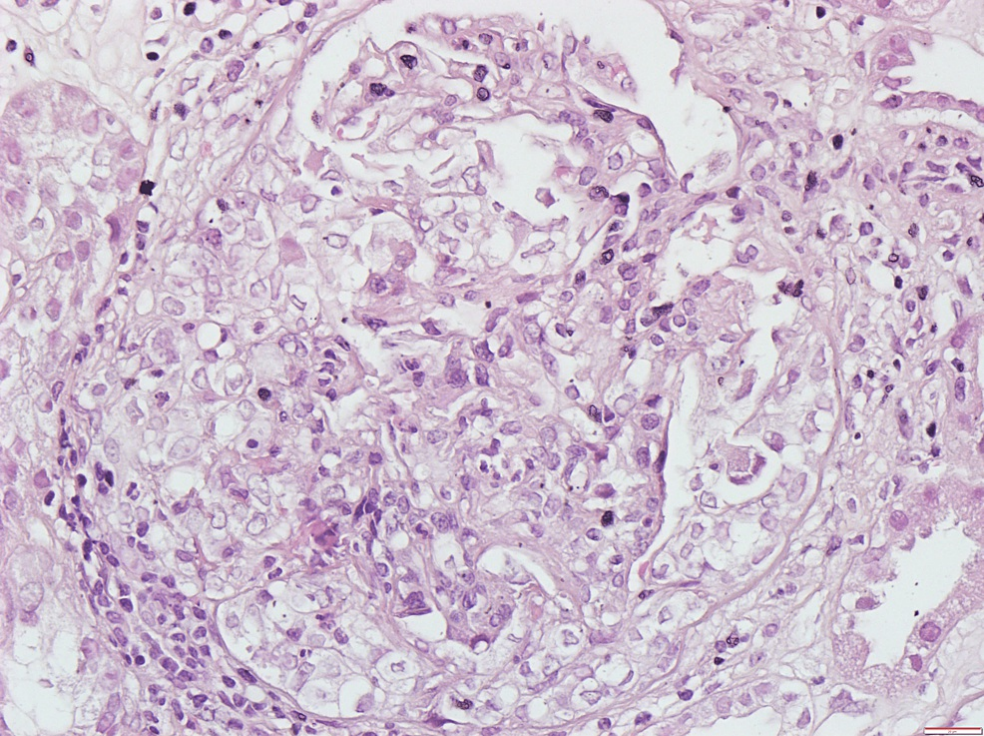
Renal biopsy histology showing the presence of one glomerulus, one cell crescent, and the presence of polymorphonuclear neutrophils in the glomerular areas.

**Figure 3:**
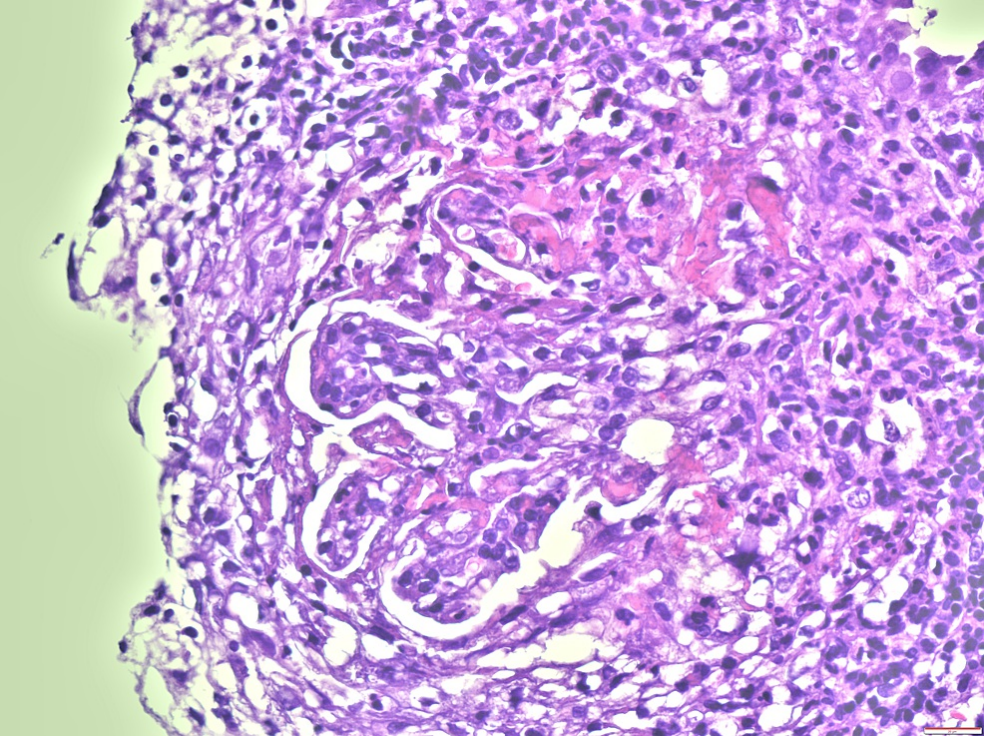
Renal biopsy histology showing the presence of one glomerulus with fibrinoid necrosis of glomerular loops and polymorphonuclear neutrophils.

Etiological investigation revealed negative blood cultures and urine cultures, a urine protein to creatinine ratio of 8 mg/mg, urinary sediment with erythrocyturia, and a high sedimentation rate with ANCA-PR3 positivity > 1067 U/ml (as seen above in Table 1). Renal biopsy demonstrated necrotizing crescentic glomerulonephritis compatible with a pauci-immune etiology mediated by ANCA-PR3 (Figures 2, 3). Therefore, a diagnosis of GPA with pulmonary, renal, and ear involvement was made. Immunosuppressive therapy was then initiated involving corticotherapy pulses with methylprednisolone, followed by the daily administration of prednisolone at a dose of 1 mg/kg/day, two doses of 1 g rituximab, and two doses of 1 g cyclophosphamide.

There was a favorable, sustained clinical and analytical improvement after the initiation of therapy, with a progressive reduction and even suspension of supplementary oxygen therapy without the need for new sessions of renal replacement. The creatinine value remained stable at 4 mg/dL. The patient was discharged on the 32nd day of hospitalization with a prescription of 40 mg/day prednisolone and a re-evaluation appointment as an internal medicine and nephrology outpatient.

One week after discharge, the patient developed generalized purpuric lesions without associated pruritus, which were interpreted as the cutaneous involvement of ANCA-PR3 vasculitis. The dose of prednisolone was increased to 60 mg/day, which resulted in a lessening of the lesions.

During the outpatient follow-up appointment, the patient received another pulse of 1 g cyclophosphamide. Subsequently, the dose of corticotherapy was gradually reduced, and maintenance therapy was undertaken with 1 g of rituximab every six months. Throughout a year of follow-up, there was an improvement in the blood count and erythrocyte sedimentation rate. Creatinine remained stable at 3 mg/dl, urinary sediment maintained slight erythrocyturia and proteinuria, and the ANCA-PR3 value was negative. The patient exhibited a normalization of the alterations previously visible in chest imaging examinations, with clinical improvement and no recurrence of symptoms. The patient currently maintains regular follow-up outpatient appointments.

## Discussion

The presented case describes a form of GPA with pulmonary, renal, cutaneous, and ear involvement. This disease typically presents with constitutional symptoms, such as fatigue, anorexia, weight loss, fever, and myalgia, which may have been present for several months before diagnosis [1,4]. In GPA, the lungs are commonly involved. Necrotizing granulomatous lung lesions may translate into cavitations or nodular lesions observed in chest CT, as demonstrated in this clinical case. There may also be upper respiratory system involvement, which may manifest as rhinitis, sinusitis, or otitis media. Hearing loss, scleritis, or uveitis may also occur. Purpuric lesions in the lower extremities or nodular skin lesions are also common. Mesenteric vasculitis may manifest with abdominal pain and hematozoa. Occasionally, hepatic, pancreatic, cardiac, or central nervous system involvement occurs. Renal disease is a common manifestation of ANCA-associated vasculitis and is an important predictor of mortality. The typical renal presentation is that of rapidly progressive glomerulonephritis with reduced renal function accompanied by subnephrotic proteinuria, microscopic hematuria, and hypertension [4,5].

Treatment is based on immunosuppression and is divided into two stages. The first is an initial induction phase (the first three to six months), intended to rapidly suppress the inflammatory process and minimize tissue damage. The second is a maintenance phase (24-48 months) that aims to prevent the disease’s recurrence. Therapeutic advances have transformed ANCA vasculitis into a chronic disease. However, relapse is common, occurring in 30-50% of patients within the first five years, often 12-18 months after the suspension of immunosuppression. The risk factors for relapse are ANCA-PR3, the GPA phenotype, preserved renal function, and the involvement of the ear, nose, or upper airway [1,3].

In this case, auditory involvement may have been the first manifestation of the disease, followed by pulmonary, renal, and later cutaneous involvement. The detection of ANCA-PR3 was fundamental, allowing for diagnosis before renal biopsy results and the establishment of targeted therapy.

The early initiation of immunosuppressive therapy is fundamental for preventing the progression of kidney damage, regardless of the glomerular filtration rate at presentation. The remission of kidney disease is defined by the stabilization or improvement of the serum creatinine value and the resolution of hematuria [3].

This patient is still being followed up at outpatient appointments. Due to the high risk of recurrence, close monitoring and surveillance are essential.

## Conclusions

Granulomatosis with polyangiitis remains a severe and chronic disease with a high risk of recurrence. However, with appropriate long-term treatments, its prognosis has improved. The authors describe a patient with auditory symptoms as the earliest GPA manifestation, demonstrating the clinical challenge. It is important for clinicians to recognize the wide variety of GPA presentations as early recognition will help in timely diagnosis and appropriate treatment, reducing associated morbidity and mortality.
